# A network analysis of alexithymia, empathy, and suicidal ideation in Chinese adolescents with major depressive disorders

**DOI:** 10.3389/fpsyt.2025.1543651

**Published:** 2025-04-16

**Authors:** Yudong Shi, Qing Zhang, Song Wang, Ling Zhang, Zhiwei Liu, Jiawei Wang, Xiangfen Luo, Xiangwang Wen, Huanzhong Liu

**Affiliations:** ^1^ Department of Psychiatry, Chaohu Hospital of Anhui Medical University, Hefei, Anhui, China; ^2^ Anhui Psychiatric Center, Anhui Medical University, Hefei, Anhui, China; ^3^ Department of Psychiatry, Fuyang Third People’s Hospital, Fuyang, Anhui, China; ^4^ Department of Psychiatry, Bozhou People’s Hospital, Bozhou, Anhui, China; ^5^ Department of Psychiatry, The Second Affiliated Hospital of Bengbu Medical College, Bengbu, Anhui, China; ^6^ Department of Psychiatry, Ma’anshan Fourth People’s Hospital, Ma’anshan, Anhui, China

**Keywords:** major depressive disorder, alexithymia, adolescents, empathy, network analysis, suicide ideation

## Abstract

**Background:**

Suicidal ideation is prevalent in major depressive disorder (MDD) and is closely related to empathy and alexithymia. While traditional approaches (e.g., regression models) focus on linear associations, network analysis provides unique advantages by mapping dynamic symptom interactions and identifying pivotal nodes that may drive suicidal risk. This study investigates these relationships through a network lens to reveal actionable intervention targets.

**Methods:**

The study included 329 adolescents with MDD (ages 12–18). The Alexithymia Scale (TAS-20), Interpersonal Reactivity Index (IRI), and the Positive and Negative Suicide Ideation scale (PNSI) were used to assess alexithymia, empathy, and suicidal ideation levels, respectively. Network analysis was conducted to model the relationships between symptoms and calculate centrality and stability indices.

**Results:**

Network analysis revealed strong stability with Emotional Identification Difficulty (DIF) and Personal Distress (PD) identified as the most influential core symptoms, exhibiting the strongest bridging roles between emotional dysfunction and suicidal ideation. DIF showed particularly robust connections to both PD and suicidal ideation, while comparative subgroup analyses indicated no significant differences in network patterns between first-episode and recurrent MDD patients, suggesting consistent symptom dynamics across illness stages.

**Conclusion:**

By revealing DIF and PD as central therapeutic targets, this study demonstrates how network analysis can uncover intervention opportunities missed by traditional approaches. Clinically, targeting these nodes through emotion recognition training and distress tolerance interventions may disrupt the pathway to suicidality in adolescents with MDD.

## 1 Introduction

Major Depressive Disorder (MDD) is a severe mood disorder with an estimated global prevalence of approximately 229 million people, characterized by hallmark symptoms of persistent low mood and significant cognitive impairments (1). According to the Global Burden of Disease data, Depressive Disorder ranked 15th in disability-adjusted life years (DALYs) and 2nd in years lived with disability (YLDs), occupying the highest burden among mental disorders (1, 2). The reasons for this phenomenon, in addition to comorbidities between MDD and other diseases, include suicide as a significant contributing factor. The risk of suicide mortality for individuals with MDD was nearly 20 times higher compared to the general population (3). Previous studies emphasized that MDD were often accompanied by emotional dysregulation, which might have been an underlying mechanism contributing to the increased risk of suicide (4, 5). Emotion expression, identification, and empathy are crucial in initial emotional processing and play key roles in emotion regulation. When these abilities are impaired, it can lead to emotional dysregulation and may increase suicide risk in individuals with depression (6, 7).

Alexithymia is defined as a significant difficulty in recognizing, describing, and paying attention to one’s internal emotional states (8). The prevalence of alexithymia in MDD patients ranges from 40% to 70% (9, 10), significantly higher than the 12%–36% found in the general population (11–13), underscoring its significant impact on emotional regulation and its potential role in suicidal ideation. Research demonstrated that individuals with higher levels of alexithymia were more frequently associated with suicidal ideation, especially those with MDD (14, 15). Such as, an exploratory study had found that drug naïve patients with MDD were more likely to experience suicidal ideation compared to those without alexithymia (16). This phenomenon may be attributed to alexithymia leading to difficulties in emotion management, which in turn amplifies feelings of stress and helplessness in patients, intensifying thoughts of self-harm (14, 15). For example, mediation analysis indicated that alexithymia has a direct positive effect on suicidal ideation in patients with MDD and can additionally increase suicide risk indirectly by intensifying patients’ levels of stress and depression (6). Another correlational study also found that the severity of alexithymia in MDD patients was positively associated with feelings of helplessness and suicidal ideation (17). Moreover, difficulties in recognizing one’s own emotions may result in deficits in empathy toward others (18, 19). Recent research suggests that both alexithymia and empathy share common neural pathways, which could explain the interaction between these two constructs. For example, abnormalities in the connectivity between the right basolateral amygdala and the left precuneus, and the left-right asymmetry of the gyrus rectus (20, 21). Furthermore, affective temperament may also play a significant role in shaping an individual’s empathy, alexithymia, and their relationship with suicidal ideation (22). Studies have found that individuals with higher scores in cyclothymic, irritable, depressive, and anxious temperaments, or lower scores in hyperthymic temperament, tend to exhibit more severe emotional expression difficulties and empathy deficits, alongside higher levels of suicidal ideation and behavior (23–26).

In parallel with alexithymia, empathy defined as the ability to recognize and understand others’ emotions and respond appropriately is another key factor in emotional regulation and suicide risk (27). Unlike the internally emotion-oriented nature of alexithymia, empathy is externally emotion-oriented, involving a positive response to others’ emotions and comprising two components: cognitive empathy, the understanding of others’ emotional states, and emotional empathy, the resonance with others’ emotional states (28). Empathy in individuals with MDD may be impaired to varying degrees. Previous studies have indicated deficits in both cognitive and emotional empathy among MDD patients (29), while another study found a reduction solely in emotional empathy (30). Researches also suggested that although empathy decreased, depressed patients tended to experience others’ negative emotions due to a negativity bias in emotional processing (31, 32). This excessive empathetic experience of negative emotions may contribute to personal distress and emotional exhaustion, potentially fostering the development of suicidal ideation (31, 33)​. However, on the relationship between empathy and suicide in patients with MDD were currently limited. A cross-sectional survey of adult patients with depression who experienced lower cognitive empathy were associated with a higher risk of suicide (7). In contrast, no correlation was found between empathy and suicidal behavior in individuals with depression who attempted suicide (34). Additionally, research on empathy and alexithymia found that depressed patients with high levels of alexithymia scored significantly lower in emotional empathy and perspective-taking, and exhibited more personal distress. Nevertheless, the sample size was small, with only 29 participants, limiting its representativeness (35).

Overall, previous studies have primarily focused on the impact of either alexithymia or empathy deficits on suicidal ideation individually, with limited investigation into the specific connections and key factors among alexithymia, empathy, and suicidal ideation within adolescent patients with MDD. Network analysis in psychopathology, as a novel analytical approach, replaces traditional conceptualizations of mental disorders by focusing on the direct interactions between symptoms (36). This method visualizes and quantifies these connections, enabling researchers to identify central nodes or critical links that play a pivotal role in the overall network. Compared to traditional regression analysis, network analysis more effectively captures complex nonlinear relationships and interactions among multiple variables (37).

This study aims to use network analysis to elucidate the role of alexithymia and empathy disorders in suicidal ideation among patients with depression. We hypothesize that: (1) suicidal ideation is associated with both empathy and alexithymia, with a positive correlation between suicidal ideation and the personal distress dimension of empathy, as well as the difficulty in emotion recognition dimension of alexithymia; (2) there is a strong association between alexithymia and empathy; (3) the network structure will differ between first episode and non-first episode depression subgroups.

## 2 Method

### 2.1 Participants and procedure

This cross-sectional study was conducted from January to August 2021 in three general hospitals and four psychiatric hospitals in Anhui Province, China. Adolescent patients were eligible to participate if they met the following criteria: 1) diagnosed with major depressive disorder (MDD) based on a structured clinical interview according to DSM-5 guidelines; 2) aged between 12 and 18 years and of Han Chinese ethnicity; and 3) able to understand the questionnaires. Individuals with other psychiatric or neurological disorders or intellectual disabilities were excluded from the study. All enrolled patients were referred for inclusion after evaluation by trained professional psychiatrists.

Participants and their guardians gave written informed consent after being informed about the study’s objectives and procedures. The research was approved by the Medical Ethics Committee of Chaohu Hospital, Anhui Medical University (approval number: 202009-kyxm-04).

### 2.2 Materials and methods

Participants’ demographic characteristics were collected, including gender(male or female), age, body mass index (BMI), educational level and first-episode (yes or no).

#### 2.2.1 The Toronto alexithymia scale-20

The TAS-20 is a widely used scale for assessing alexithymia, consisting of 20 items rated on a 5-point Likert scale, ranging from 1 (strongly disagree) to 5 (strongly agree) (38). The scale measures three key components: Difficulty Identifying Feelings (DIF), Difficulty Describing Feelings (DDF), and Externally Oriented Thinking (EOT). Higher TAS-20 scores indicate more pronounced alexithymic traits. Studies have shown that the TAS-20 demonstrates good reliability in Chinese populations, with a Cronbach’s alpha of 0.79 (39). In this study, the scale showed strong reliability, with a Cronbach’s alpha of 0.744.

#### 2.2.2 Interpersonal reactivity index

IRI is a self-report tool used to assess an individual’s empathy and emotional responsiveness, consisting of 28 items rated on a five-point Likert scale, ranging from 1 to 5 points (28). The scale is divided into four dimensions: Fantasy (FS), Empathic Concern (EC), Personal Distress (PD), and Perspective Taking (PT). The total score ranges from 28 to 140 points. Higher scores typically indicate a stronger capacity for empathy and emotional sensitivity, suggesting that individuals are better able to understand and care for the emotional states of others. The IRI scale demonstrated good internal consistency in the Chinese population, with a Cronbach’s alpha of 0.70 (40). In this study, the scale demonstrated solid reliability, with a Cronbach’s alpha of 0.769.

#### 2.2.3 Positive and negative suicide ideation

The Positive and Negative Suicide Ideation scale (PNSI) assesses suicidal ideation through 14 items scored on a five-point Likert scale from “strongly disagree” (1) to “strongly agree” (5) (41). It consists of two dimensions: positive suicidal ideation and negative suicidal ideation, with a total score ranging from 14 to 70 points. Higher scores indicate stronger positive and negative suicidal ideations, reflecting a higher suicide risk and complex psychological state. The PNSI scale has demonstrated good internal consistency in the Chinese population, with a Cronbach’s alpha of 0.88 (42), and a strong reliability in our sample (Cronbach’s alpha = 0.938). The total PANSI score was used as an indicator of empathy.

### 2.3 Statistical analysis

#### 2.3.1 Network estimation

All analyses were conducted using RStudio version 4.4.0. The network structure was estimated using the Gaussian Graphical Model (GGM) with the Extended Bayesian Information Criterion Lasso (EBICglasso) regularization method, which serves to reduce redundant edges and increase network sparsity. The bootnet package (version 1.6.0) (43) was used to construct the network, applying Spearman correlation to calculate the relationships between variables, and the network was visualized using the qgraph package (version 1.9.8) (44), where blue edges indicate positive associations and red edges indicate negative associations. Additionally, the mgm package (version 1.2-14) was employed to perform node-wise predictions, with green circles representing the magnitude of the predicted values (45).

#### 2.3.2 Network centrality

Network centrality was assessed using the qgraph package (version 1.9.8) (44). Indicators for identifying prominent nodes in the overall network included betweenness, closeness, strength, and expected influence (EI). Given that both positive and negative edges are present in the network analyzed in this study, EI serves as a more suitable measure. Additionally, bridge centrality (bridge expected influence, BEI) was assessed using the networktools package (version 1.5.2) to highlight nodes that act as bridges between groups (46). This analysis aids in identifying significant connections between different symptom groups.

#### 2.3.3 Network accuracy and stability

The bootstrapping method was utilized to assess the accuracy and stability of the network, involving 1000 iterations with the bootnet package (version 1.6.0) (43). To evaluate the stability of network centrality, we used the case-dropping subset bootstrap approach, which systematically drops individual cases (i.e., participants) from the dataset to test whether centrality measures (e.g., EI, BEI) remain stable across different subsets of the data. This approach provides insight into the robustness of central nodes and edges in the network. The accuracy of the edges was assessed by calculating the 95% confidence interval (CI) of the bootstrapped edge weights, ensuring that we account for the variability in edge estimates across the resampling iterations. Finally, the correlation stability coefficient (CS coefficient) was calculated to assess the stability of the network’s centrality indices. A CS coefficient value above 0.5 indicates that the centrality indices are stable, meaning they are unlikely to change significantly with the exclusion of a small portion of the sample (43).

#### 2.3.4 Network comparison test

To explore differences in network structures among patients at various stages of illness, this study compared first episode (FE) and non-first episode (NFE) subgroups using the Network Comparison Test (NCT) (version 2.2.2) (47). Primary indicators comprised global strength to assess overall network strength, structure invariance for consistency across phases, and edge weights to identify differences in symptom connections.

## 3 Result

### 3.1 Descriptive statistics

As shown in **Table 1**, a total of 329 participants were included in this study, with an average age of 15.33 years. Among them, 241 were female, and 43.8% were junior high school students. There were 193 first-episode patients (58.7%) and 136 non-first-episode patients (41.3%). The mean total score for suicide ideation, IRI, and TAS were 47.79, 94.21, and 67.90, respectively.

**Table 1:** Social-demographic and clinical characteristics of Participants.

Variables	Median ± SD or N (%) N = 329
Female	241 (73.3)
Age(years)	15.33 ± 1.63
junior high school	144 (43.8)
senior high school	185 (56.2)
BMI (kg/m2)	20.74 ± 3.63
First-episode	193 (58.7)
PNSI	47.79 ± 12.89
IRI	94.21 ± 12.76
PT	22.51 ± 5.14
PD	25.70 ± 4.41
EC	22.71 ± 4.86
FS	23.28 ± 4.92
TAS	67.90 ± 9.06
DIF	25.76 ± 5.50
DDF	16.80 ± 2.67
EOT	25.34 ± 4.06

Note: BMI, Body Mass Index; PNSI, Positive and Negative Suicide Ideation; IRI, Interpersonal Reactivity Index; PD, Personal Distress; PT, Perspective Taking; EC, Empathic Concern; FS, Fantasy; TAS, The Toronto Alexithymia Scale; DIF, Difficulty Identifying Feelings; DDF, Difficulty Describing Feelings; EOT, Externally Oriented Thinking.

### 3.2 Edge weights and centrality

**Figure 1:**
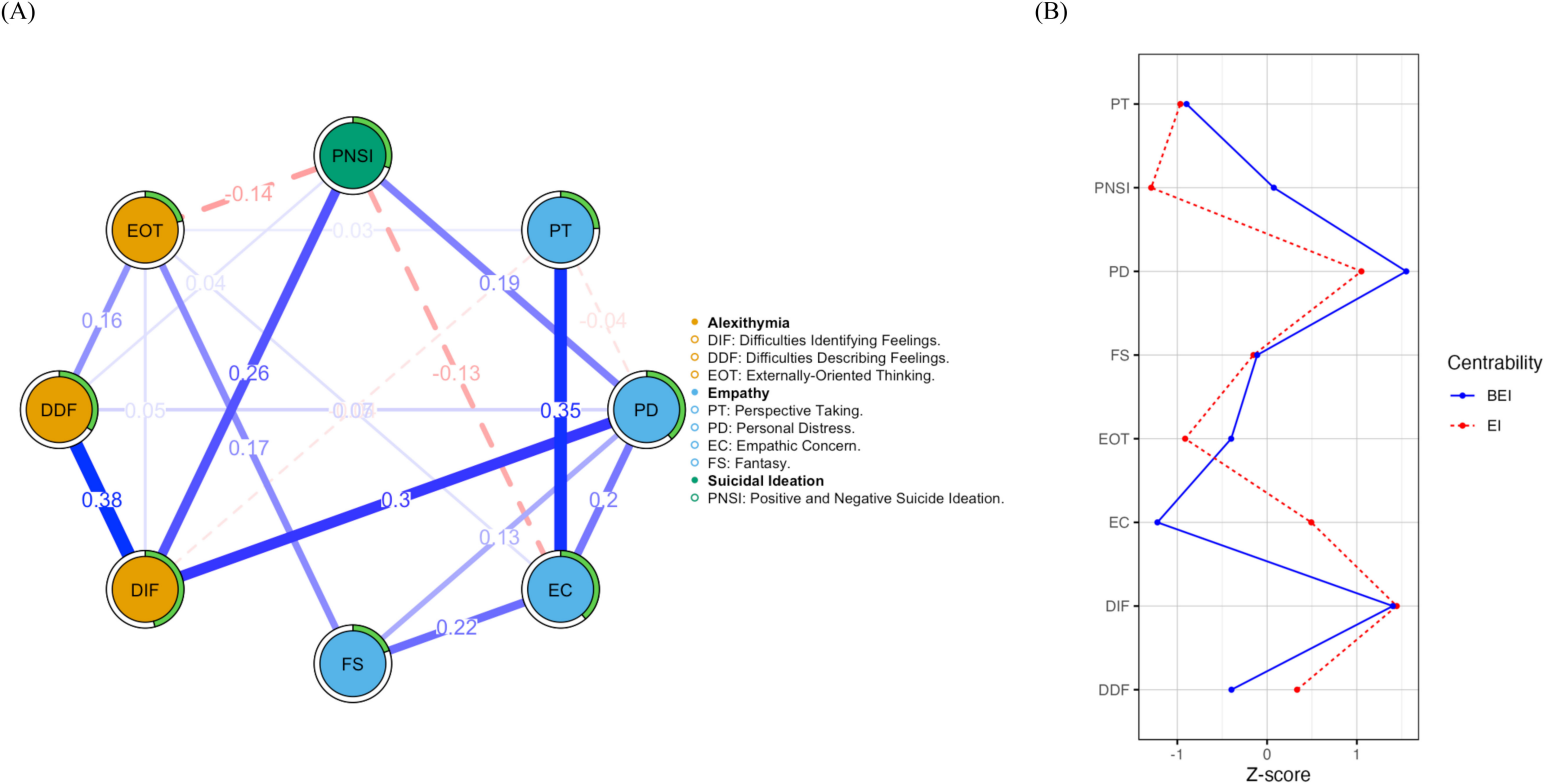
**(A)** Regularized partial correlation network for MDD patients (n = 329). Blue edges represent positive correlations, and red edges represent negative correlations. The green ring around each node indicates its predictability. Thicker edges represent stronger partial correlations. **(B)** Network centrality index. Centrality is expressed as a standardized z-score, with the red line representing the EI index and the blue line representing the BEI index. Nodes with a high EI index have significant overall influence, affecting many other nodes, while nodes with a high BEI index act as bridges, connecting different parts of the network and facilitating communication between clusters. EI, Expected Influence; BEI, Bridge Expected Influence.

**Figure 1A** showed the overall structure of the network analysis. Overall, nodes representing various dimensions within each scale showed positive correlations, indicating strong “within-scale” edges. The network included a total of 28 edges, with 19 being non-zero edges (67.9%), indicating a densely connected structure. The highest edge weights between nodes were found between DIF and DDF (r = 0.381), PT and EC (r = 0.352), and PD and DIF (r = 0.300), while the strongest connections between scales were observed between PD and DIF, and DIF and PNSI (0.258). Additionally, negative correlations were found between EOT, EC, and PNSI. The size of edge weights and the significance of differences between them were shown in **Supplementary Table S1**, **Supplementary Figure S1**.

**Table 2**, **Figure 1B** presented the specific values and visualization results of network centrality metrics EI and BEI for different nodes. The strongest EI node was DIF (0.95), followed by PD (0.84). For BEI, the strongest nodes were PD (1.55) and DIF (1.40). **Supplementary Figures S3**, **S4** showed that the bootstrap difference test results indicated statistically significant differences between DIF, PD, and the remaining nodes. Additionally, DIF (0.39) and PD (0.46) had the highest predictability values. The results suggested that DIF and PD occupied central positions in the network, with the greatest influence on the other nodes in the network and the highest predictability by the remaining nodes.

**Table 2:** List of Nodes and Centrality Metrics.

Nodes	EI	BEI	Predictability (R2)
PNSI	0.22	0.07	0.30
PT	0.30	-0.90	0.24
PD	0.84	1.55	0.39
EC	0.69	-1.22	0.39
FS	0.52	-0.11	0.20
DIF	0.95	1.40	0.46
DDF	0.65	-0.40	0.34
EOT	0.32	-0.40	0.21

EI, Expected Influence; BEI, Bridge Expected Influence; PNSI, Positive and Negative Suicide Ideation; IRI, Interpersonal Reactivity Index; PD, Personal Distress; PT, Perspective Taking; EC, Empathic Concern; FS, Fantasy; TAS, The Toronto Alexithymia Scale; DIF, Difficulty Identifying Feelings; DDF, Difficulty Describing Feelings; EOT, Externally Oriented Thinking.

### 3.3 Network stability and group comparison

**Figure 2:**
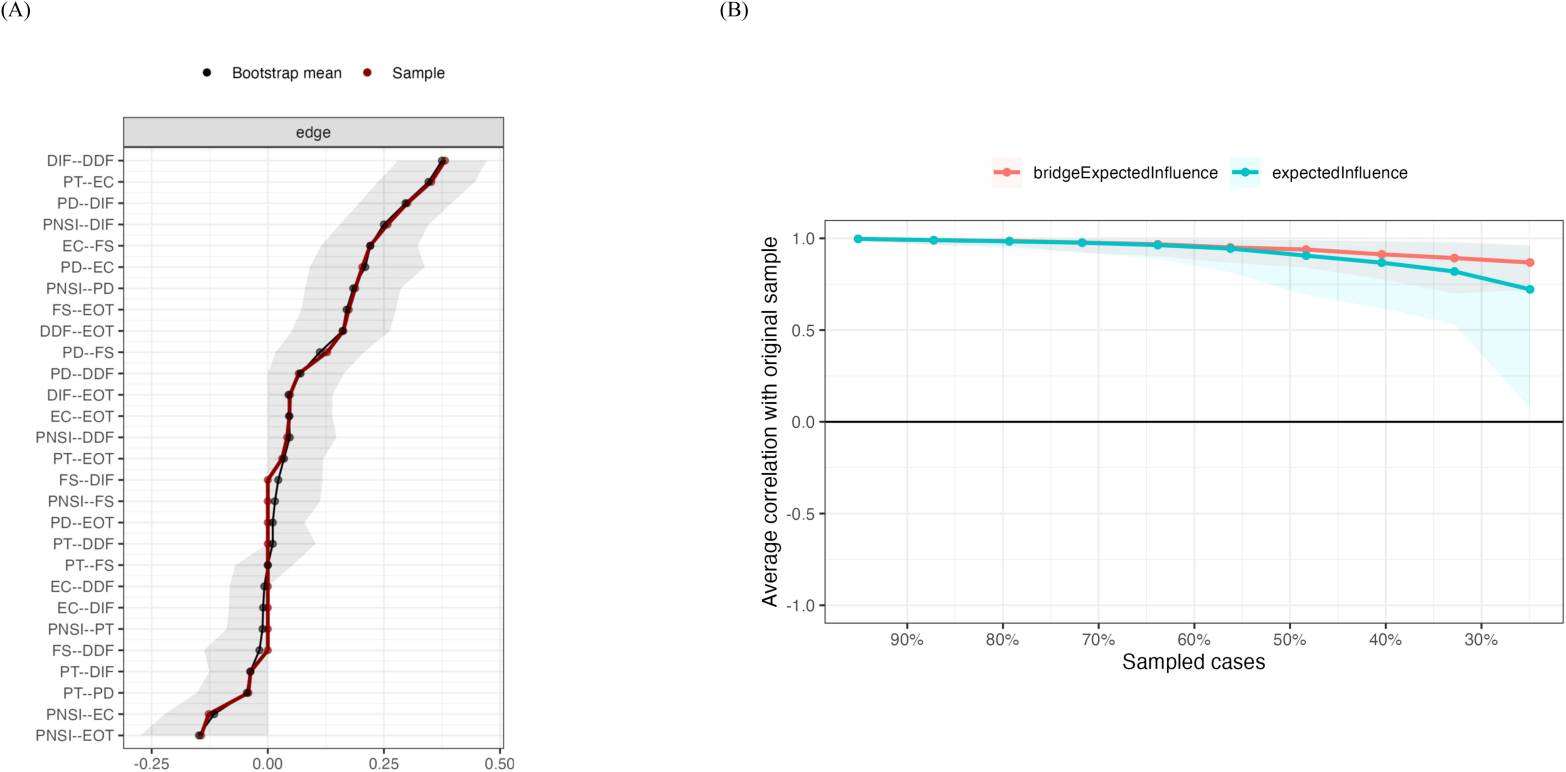
**(A)** Bootstrap Stability Test of Edge Weights. **(B)** Bootstrap Stability Test of Network Bridge and Expected Influence. DIF, Difficulty Identifying Feelings; DDF, Difficulty Describing Feelings; EOT, Externally Oriented Thinking; PD, Personal Distress; PT, Perspective Taking; EC, Empathic Concern; FS, Fantasy; PNSI, Positive and Negative Suicide Ideation.

Nonparametric bootstrap analysis of edge accuracy indicated that edge precision was acceptable, as shown in **Figure 2A**. The narrow gray areas represent the confidence interval (CI) range, with smaller CIs indicating more accurate edge estimates. The near overlap of the brown and black lines further suggests high consistency between the bootstrap edge means and the sample edge weights. Additionally, stability analysis of EI and BEI using case-dropping subset bootstrap showed that, although the correlation between centrality indices and those of the full sample slightly decreased as subset size reduced, overall stability remained high (**Figure 2B**). Finally, the CS coefficients confirmed this stability, with EI and BEI yielding CS coefficients of 0.596 and 0.672, respectively.

The network analysis visualization and centrality indices for the NFE and FE subgroups are shown in **Supplementary Figures S4** and **S5**. Comparisons of network estimates between subgroups revealed no statistical differences in global strength invariance (S = 0.52, p = 0.65), structural invariance (M = 0.19, p = 0.75), or edge weights.

## 4 Discussion

To our knowledge, this study was the first to apply network analysis to examine the complex associations among alexithymia, empathy, and suicidal ideation in adolescent patients with MDD. The primary findings are as follows. First, the network identified central nodes for alexithymia and empathy, particularly DIF and PD. Second, the strongest edge weight between symptom clusters was found between DIF and PD. Finally, network comparison analysis showed no significant structural differences between first-episode and recurrent MDD patients, suggesting a stable association structure among alexithymia, empathy deficits, and suicidal ideation across illness stages.

This study identified DIF as the strongest EI indicator and the most predictable node in the integrated network analysis of alexithymia, empathy, and suicidal ideation, underscoring its role as a core symptom of alexithymia with substantial direct impact on other nodes. According to emotion regulation theories, accurately identifying one’s emotions is foundational for selecting effective regulation strategies (48). Individuals with alexithymia were often positively associated with emotional dysregulation and tend to employ fewer adaptive emotion regulation strategies (e.g., cognitive reappraisal, problem-solving, and seeking social support) and more maladaptive strategies (e.g., emotional suppression, behavioral withdrawal, and neglect) (49). Similar studies have also found that DIF was directly positively correlated with anxiety and stress, and that it indirectly promoted the development of anxiety and depression through the suppression of reappraisal (50). This deficiency increases susceptibility to negative thoughts and actions, such as suicidal ideation and behaviors, under emotional stress, aligning with our finding of a positive association between DIF and suicidal ideation (6).The direct link between DIF and suicidal ideation in MDD patients has also been confirmed in previous studies, which have found a weaker or non-existent relationship between DDF and suicidal ideation. This phenomenon further supports the critical role of DIF within the overall network (6, 17). Currently, there is a lack of longitudinal studies examining the impact of treatment for alexithymia in patients with MDD on empathy and suicidal ideation. Previous research has shown that deep brain stimulation, art therapy, and clay art therapy can reduce the severity of emotional expression disorders in patients with schizophrenia, depression, and Parkinson’s disease (51–53). However, the effects on the depressive population and other nodes within the network still require further validation.

Furthermore, we found a significant positive correlation between DIF and PD, reflecting a potential link between emotional processing deficits and self-related emotional responses in empathy. Recent studies have revealed that, beyond the traditional definition of alexithymia, which focuses on internal emotional cognition deficits, individuals with this condition also exhibit impairments in recognizing and recalling external emotional information (54–56). Through facial expression recognition and recognition memory tests of real social videos, individuals showed a significant decrease in the accuracy of immediate recognition and recall of emotional expressions (relative to neutral expressions) in social activities, which was significantly negatively correlated with DIF (54). The foundation of empathy lies in the accurate perception of others’ emotions, so it is reasonable to assume that alexithymia is related to impaired empathy. In fact, this connection has been demonstrated in both the general population and individuals with mental disorders, particularly in autism (35, 57, 58). For example, studies on individuals with autism have shown that alexithymia impairs empathy, primarily manifesting as a decrease in emotional empathy and an increase in PD (57). Similarly, a study on a small sample of individuals with MDD found that, compared to the low alexithymia group, the high alexithymia group showed a decline in cognitive empathy and higher levels of PD (35). The strong correlation between alexithymia and empathy could be due to overlapping neural mechanisms, particularly in brain areas like anterior insula and the anterior cingulate cortex, which play pivotal roles in the shared processing of self and others’ emotions (59–61).

More importantly, PD serves as the variable with the highest cross-symptom group node (BEI) in the network, demonstrating a significant impact on suicidal ideation. Most studies have shown that patients with MDD often experience emotional exhaustion, primarily caused by specific negative cognitive biases, characterized by a reduced responsiveness to positive emotions and heightened sensitivity to negative emotions (31, 32, 62). Compared to healthy controls, individuals with MDD show stronger alter centric biases, being more influenced by others’ negative emotions, related to emotional contagion and increased PD (62). This empathy impairment may be related to deficits in executive function among patients with MDD. A meta-analysis revealed a strong association between empathy and executive function, with inhibitory control serving as a predictor of emotional empathy (63). Deficits in inhibitory control among MDD patients suggest that when empathizing with others, decreased emotional empathy was accompanied by reduced ability to regulate emotional contagion, leading to increased personal distress (63, 64). Additionally, studies suggest that disruptions in the excitation/inhibition balance of the anterior cingulate cortex and medial prefrontal cortex underlie the empathy impairments observed in MDD (65). The distress resulting from empathy impairment was thought to be linked to suicide (34, 66), consistent with our findings that PD was positively associated with suicidal ideation. PD, as a potential mediating node between alexithymia and suicidal ideation, underscores the importance of considering the entire network in interventions, rather than focusing solely on the effects of individual nodes such as alexithymia or empathy. This highlights the importance of personalized interventions in adolescent mental health care, while systemic mental health care can effectively manage adolescent patients and ensure continuity of care (67, 68).

Notable, the group analysis in this study did not reveal significant differences in network structure between FE and NFE patients, suggesting that the overall network model may be stable across different stages of depression, unaffected by the progression of the illness. This finding is of significant importance as it supports the idea of implementing unified interventions for adolescent depression patients, particularly those focusing on improving emotional recognition and regulation abilities, which may have positive effects regardless of the stage of illness. Due to the higher prevalence of depression in females, the proportion of females in this study was significantly higher than that of males (69). Studies have found that there are sex differences in the emotion regulation strategies used; for example, females may be more likely to use mindfulness, reflection, or emotional expression to cope with negative emotions, while males may tend to use suppression or avoidance strategies (70, 71). Additionally, female patients with depression exhibit significantly higher levels of emotional empathy and suicidal ideation compared to males (72, 73). Therefore, the underlying mechanisms linking these factors may differ by sex suggesting that the conclusions of this study may be more applicable to female populations with depression.

The limitations of this study are as follows: First, the sample was limited to adolescent depression patients, which may restrict the generalizability of the findings to other populations or age groups. Second, due to the cross-sectional design, we were unable to explore causal relationships. While network analysis revealed associations between variables, the directionality and causality of these relationships remain unclear. Longitudinal studies would be beneficial in uncovering the causal pathways between the variables and providing a more in-depth understanding of these relationships. Third, the network was constructed using self-reported measures, which are subject to recall bias and social desirability effects. These biases may compromise the accuracy of participants’ responses, potentially influencing the findings. Future studies could address this limitation by integrating biological measures or behavioral observations to complement the self-report data. Finally, the underrepresentation of male participants may have limited the ability to examine sex differences in alexithymia and suicidal ideation fully, suggesting the need for future studies with a larger male sample to investigate these potential differences.

## 5 Conclusion

This study, employing network analysis, underscores the intricate relationships among alexithymia, empathy, and suicidal ideation in adolescents with MDD. DIF was found to be closely associated with PD and suicidal ideation, emphasizing its pivotal role in emotional processing and interpersonal distress. Additionally, PD, as the strongest bridging node, serve as a mediator between alexithymia and suicidal ideation. The consistent network structure across FE and NFE patients with MDD suggests the stability of these associations across different stages of the illness. These findings highlight the importance of targeted interventions aimed at key points, such as improving emotional recognition, to alleviate emotional distress and reduce suicidal ideation. Moreover, they offer valuable directions for future longitudinal studies to investigate the causal pathways between these variables. Such research would allow for a deeper exploration of how these factors evolve and interact over time, thus enhancing our understanding of the temporal dynamics and potential causal relationships in MDD.

## Data Availability

The raw data supporting the conclusions of this article will be made available by the authors, without undue reservation.
