# Supplementary material for: A network analysis of alexithymia, empathy, and suicidal ideation in Chinese adolescents with major depressive disorders
Source: Front Psychiatry. 2025 Apr 16;16:1543651. doi: 10.3389/fpsyt.2025.1543651 (PMC12040915; doi:10.3389/fpsyt.2025.1543651)
Supplement: Supplementary file 1 [file DataSheet1.docx]

**Table S**1**.** Edge Weights in the Network of Empathy, Alexithymia, and Suicidal Ideation among Patients with MDD

**Figure S1.** Bootstrapped difference test for edge weights.

**Figure S2**. Bootstrap test of the difference in expected influence of nodes in the network.

**Figure S3**. Bootstrap test of the difference in bridge expected influence of nodes in the network.

**Figure S4**. Regularized partial correlation network for the first episode depression subgroup and non-first episode depression subgroup.

**Figure S5.** Comparison of network centrality indices between first episode and non-first episode patients.

**Table S1. Edge Weights in the Network of Empathy, Alexithymia, and Suicidal Ideation among Patients with MDD.**

| **Edge** | **Partial correlation coefficient** | **Edge** | **Partial correlation coefficient** |
| --- | --- | --- | --- |
| DIF--DDF | 0.381 | PD--DDF | 0.067 |
| PT--EC | 0.352 | DIF--EOT | 0.048 |
| PD--DIF | 0.300 | EC--EOT | 0.046 |
| PNSI--DIF | 0.258 | PNSI--DDF | 0.042 |
| EC--FS | 0.220 | PT--PD | -0.042 |
| PD--EC | 0.204 | PT--DIF | -0.037 |
| PNSI--PD | 0.188 | PT--EOT | 0.031 |
| FS--EOT | 0.174 |  |  |
| DDF--EOT | 0.163 |  |  |
| PNSI--EOT | -0.144 |  |  |
| PD--FS | 0.128 |  |  |
| PNSI--EC | -0.127 |  |  |

Note：Only non-zero edges calculated for the EBICglasso network were included, accounting for 67.9% of all edges.

**Figure S1.** Bootstrapped difference test for edge weights.


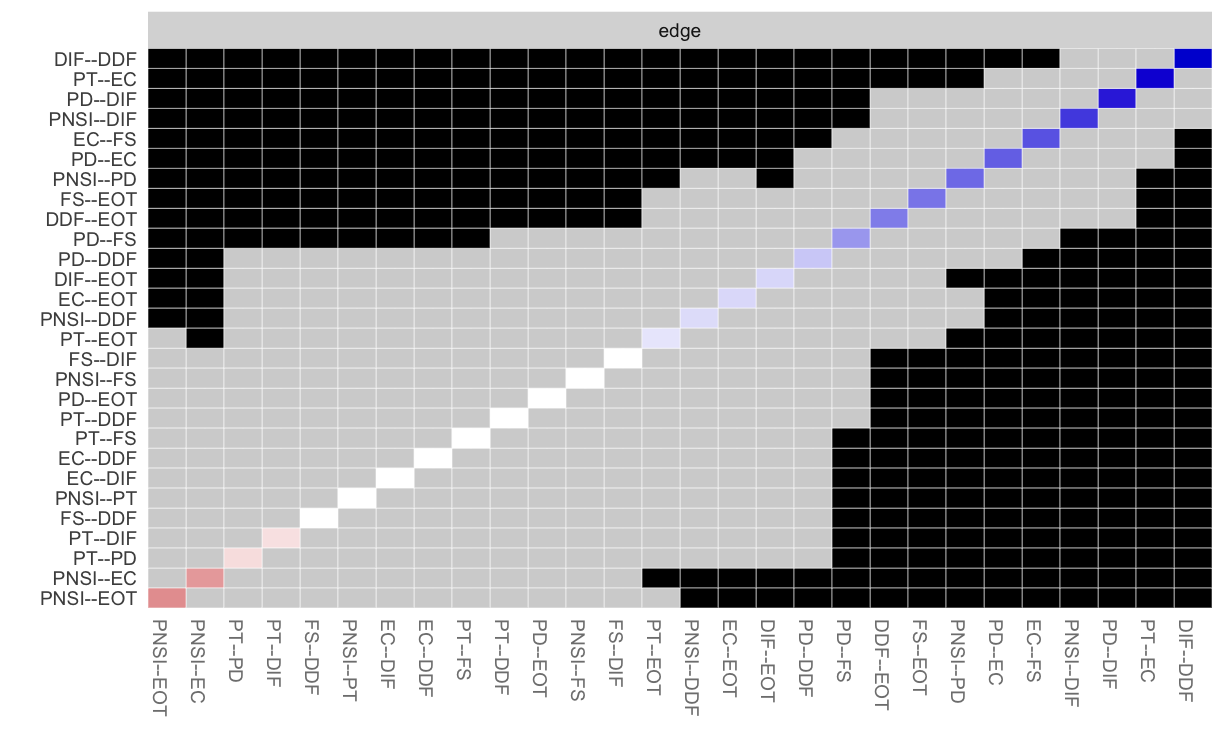


Note: Black boxes represent significant differences in expected influence between two variables, while grey boxes indicate non-significant differences.

**Figure S2**. Bootstrap test of the difference in expected influence of nodes in the network.


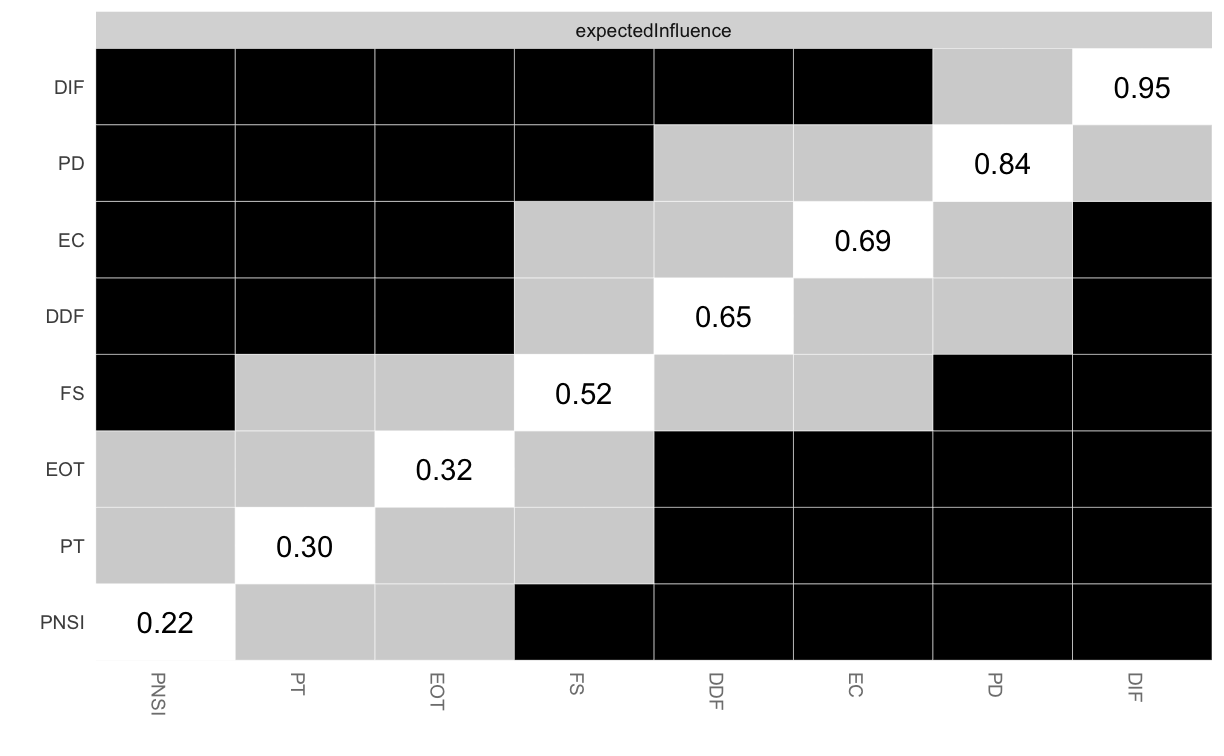


Note: The numerical magnitude of each node’s expected influence is indicated in the diagonal boxes. Black boxes represent significant differences in expected influence between two variables, while grey boxes indicate non-significant differences.

**Figure S3**. Bootstrap test of the difference in bridge expected influence of nodes in the network.


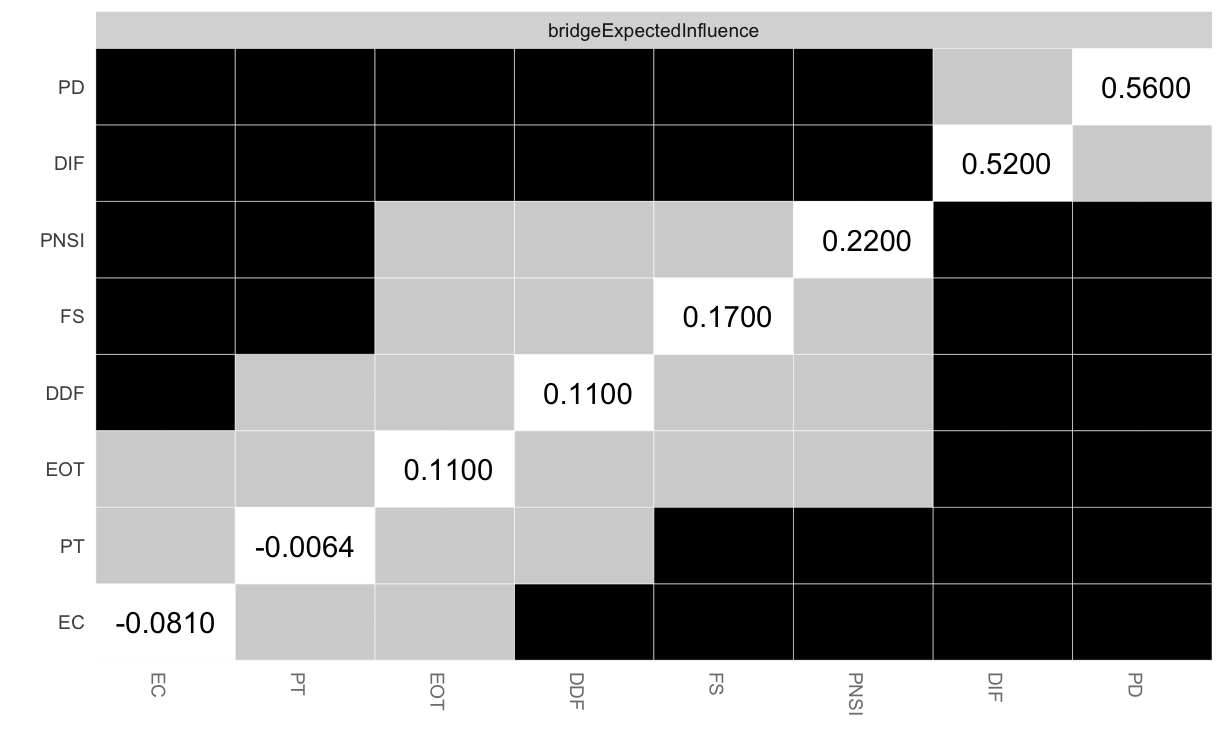


Note: The numerical magnitude of each node’s bridge expected influence is indicated in the diagonal boxes. Black boxes represent significant differences in expected influence between two variables, while grey boxes indicate non-significant differences.


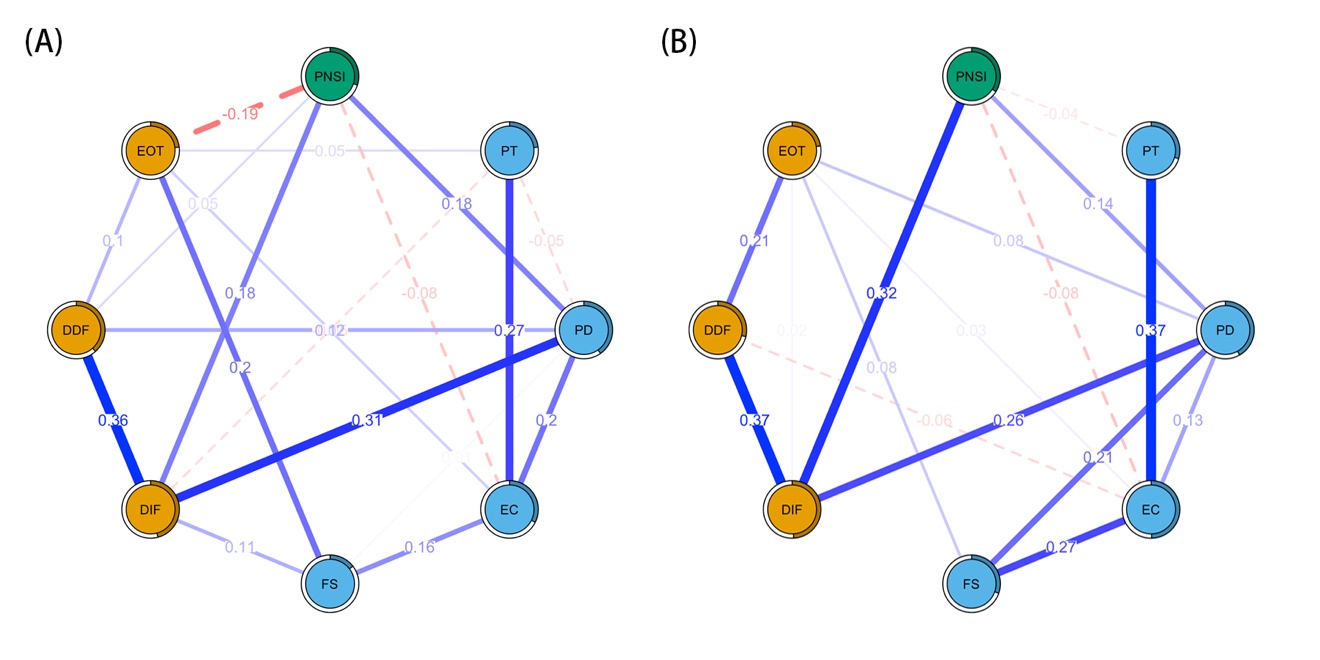


**Figure S4.** A. Regularized partial correlation network for the first episode depression subgroup (n = 193); B. Regularized partial correlation network for the recurrent depression subgroup (n = 136). Blue edges represent positive correlations, and red edges represent negative correlations. The green ring around each node indicates its predictability. Thicker edges represent stronger partial correlations. DIF, Difficulty Identifying Feelings; DDF, Difficulty Describing Feelings; EOT, Externally Oriented Thinking; PD, Personal Distress; PT, Perspective Taking; EC, Empathic Concern; FS, Fantasy; PNSI, Positive and Negative Suicide Ideation.

**Figure S5. Comparison of network centrality indices between first episode and non-first episode patients.**

**
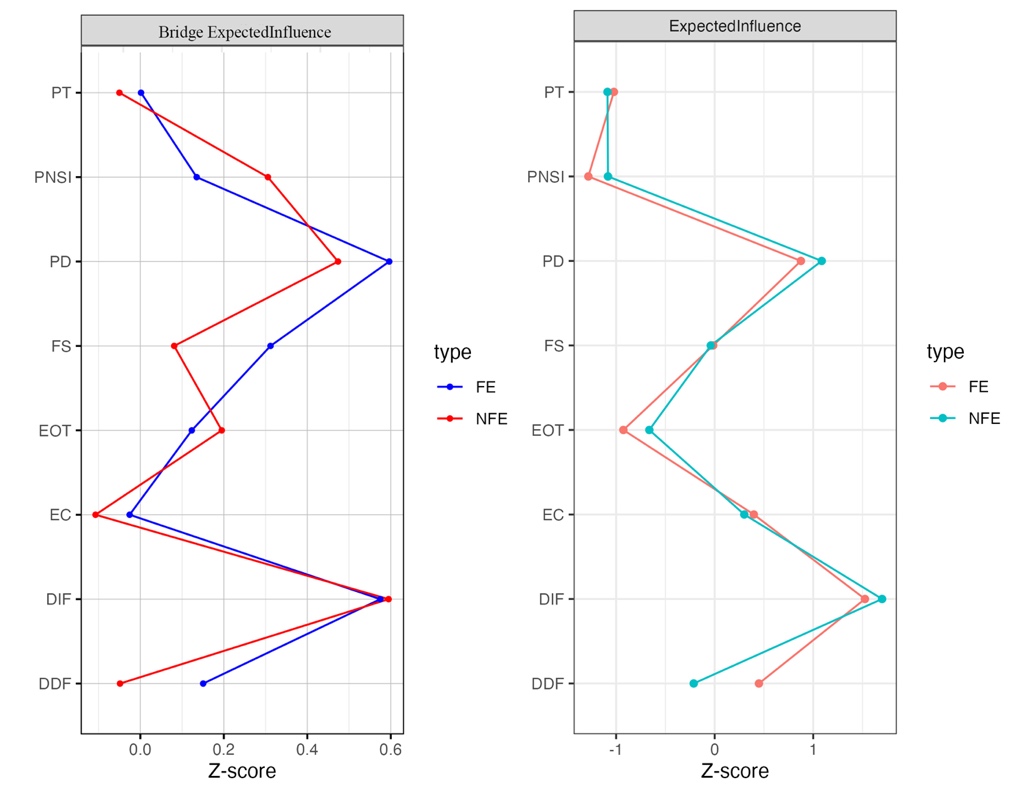
**
